# Proceedings of the Alabama State Medical Association

**Published:** 1872-04

**Authors:** Wm. Abram Love


### PROCEEDINGS OF THE ALABAMA STATE MEDICAL ASSOCIATION.

REPORTED BY WM. ABRAM LOVE, M.D.,

The annual meeting of the Alabama State Medical Association was held in the court-house at Huntsville, Alabama, commencing on the 26th of March, 1872. The Association was called to order at twenty minutes to eleven o'clock A.M., by the President, Dr. T. C. Osborn, and the Convention opened with prayer by Rev. Dr. Ross.

The roll was called by the Secretary, Dr. Jerome Cochran, of Mobile, when the following gentlemen responded to their names:

Drs. W. H. Anderson, L. S. Bonner, H. W. Bassett, J. S. Bankshead, D. E. Berger, Jerome Cochran, O. L. Crampton,

C. A. Crow, I. J. Dement, L. W. Despretz, Wm. Despretz, E. P. Gaines, J. T. Gilmore, J. R. Hoffman, Wm. Henderson, W. C. Hicklan, F. Jordan, M. H. Jordan, W. C. Jackson, G. A. Ketchum, G. E. Kumpè, James Kyle, J. S. Lynch, John Little, Jr., C. H. Mastin, T. A. Means, G. D. Norris, T. C. Osborn, N. D. Richardson, F. A. Ross, L. H. Sadler, L. M. Sheppard, D. E. Smith, T. O. Summers, J. S. Weatherly.

The Association was welcomed to Huntsville by Dr. J. J. Dement—on the part of the Madison County Medical Society and the citizens of Huntsville—in a feeling and appropriate address.

The President, Dr. T. C. Osborn, then delivered his address, which was well received, and every way characteristic of the man. Throughout, it was replete with interest to the profession of the State of Alabama, and, in very many particulars, not wanting in interest to the profession at large, particularly of the Southern States. We regret that we are unable to quote many parts of the address *verbatim.* Such being impracticable, we can but allude to some of the leading points made, and give a synopsis in brief; to attempt more than this, would do injustice to the speaker.

On the subject of compensation for services rendered by medical men to freedmen, etc., in the employ ot landlords, he dwells at some length, and is outspoken in condemnation of the present *class legislation*, and the discrimination *against* the medical profession of the State. He speaks feelingly, and without reserve, of the injustice of such laws, and the inability of the profession to have them repealed, and others more just in their provisions enacted in their stead, without resort to means which would be considered dishonorable. He calls on the profession of the State to unite as one man, and bring their moral influence to bear in the proper direction to obtain their rights before the law. Believes that the influence of the profession should be concentrated and united to elect to the General Assembly men whose influence would lead to the passage of such enactments as would protect the rights of the profession, and repeal such laws as now discriminate against them. He admonishes the profession, and pledges himself, "*to persevere unflaggingly,*

*leaving no resource untried that is at all likely to crown such efforts with success."* Recommends the passage of a law giving to the physician a lien, for fees, next to landlords and laborers. In alluding to the existing laws protecting landlords, he regards such class legislation—as the work of those interested—and points out the propriety and necessity for battling against such "*class legislation*," and instituting legislation "*for the good of the people at large.*"

Next in order is considered the subject of a "*Benevolent Aid Association*," on the plan of an insurance company, for the benefit of the widows and orphans of physicians dying in the State of Alabama. After reviewing the plan and referring to the originators (Dr. Despretz and others, of Tuscumbia, we believe), he commended it to the favorable consideration of the medical men of the State.

*Medical education* claims the attention of the President next, and he advocates a higher standard as the only means of elevating the profession of this country. At the very foundation of the difficulty in the rise and progress of the profession, as a class, is cited the *admission of students into private offices of physicians without proper preliminary or preparatory education.* Some time is devoted to this subject; and in conclusion, Dr. Osborn, in a very feeling and earnest manner, admonishes the profession to receive no students into their offices who have not attained a sufficient preliminary education to enable them to devote their time profitably to the study of medicine in all its branches.

The subject of subordinate *District Medical Associations,* consisting of the profession of several adjacent counties or convenient towns and villages, is discussed, and the formation of such associations advised as one means of uniting the profession in district centers, where they may work together for the common good of the profession and the benefit of our science.

In conclusion, the President alludes to the periodic return of *severe malarial epidemics,* seemingly with some degree of regularity, and advised the adoption of means to collect such data as would lead to a correct history of the prevalence of such epidemics.

The proposed *new Constitution* is alluded to, and the matter referred to the calm and deliberate attention and consideration of the Association.

The entire address was a regular chain of closely-linked, practical points, brought to the attention of the Association for consideration, and it is with much regret that we are unable to do justice to the speaker or the production in this brief synopsis. We have simply alluded to the leading points considered and brought to the attention of the medical men of Alabama.

The address of the President was, on motion, referred to a committee, consisting of Drs. H. W. Bassett, M. H. Jordan, and John Little, Jr.

A communication from Dr. Michel was read by Dr. J. S. Weatherly.

The report of the Secretary, Dr. Jerome Cochran, was read, received and approved.

The report of the Treasurer, Dr. W. C. Jackson, was read, received and approved.

Dr. G. E. Kumpè, Chairman of the committee to whom was referred the proposed new Constitution, asked further time, until to-morrow, to make his report—in consequence of the absence of the other members of the committee—which was granted.

The Committee on Asylum for Inebriates was called. No report, and the committee was continued.

The Committee on Memorial to the Legislature submitted a verbal report, by Dr. J. S. Weatherly, to the effect that the memorial had been prepared and presented to the Legislature, but without results. Committee continued.

The Committee on Marine Hospital reported verbally, by Dr. J. S. Weatherly, that no action had been taken. The committee were of the opinion that nothing could be done in the matter. The President, Dr. Osborn, here presented a letter from Dr. Heustiss, stating that he had heard that the United States do not desire to sell or dispose of the Hospital. Committee continued.

Committee on National Medical College was called. No report, and the committee was discharged.

Motions and resolutions being in order, Dr. Jerome Cochran read a paper on public hygiene, and the organization of a State board of health.

Dr. Weatherly read a statement of what had been done, submitting a somewhat different plan, after which some discussion followed—participated in by Drs. Cochran and Weatherly—when, on motion of Dr. Gaines, both plans were referred to a committee, to report Thursday morning. Committee—Drs. Gaines, Means, and Sheppard.

A resolution was presented—sent in by Dr. Michel—to change the time of holding the annual meeting of the Association, which was laid on the table.

Adjourned to meet for business at ten o'clock A.M. to-morrow, and at half-past seven to hear the annual oration by Dr. J. S. Weatherly.

The oration of Dr. Weatherly was unique and original in thought. We did not have the pleasure of hearing it; therefore, can not give even a synopsis. The subject was, "Woman's Rights, Viewed from a Medical Stand-Point." The Doctor acquitted himself in a becoming manner, and we have to regret that we were unable to attend on the occasion.

---

HUNTSVILLE, ALABAMA, March 27, 1872.

The Association met at fifteen minutes past ten o'clock, and was called to order by the President, Dr. T. C. Osborn. The minutes of yesterday were read and approved.

A communication from Dr. G. M. McDowell, President of the Georgia Medical Association, was read, accrediting Dr. Wm. Abram Love, of Atlanta, as a Visiting Delegate to the Alabama State Medical Association.

On motion of Dr. Gaines, a committee of three was appointed to receive Dr. Love, and introduce him to the Association. Committee—Drs. Gaines, Bassett, and Little.

The committee presented Dr. Love to the Association, when he was welcomed by the President in a cordial manner, which was duly acknowledged by Dr. Love as a compliment, extended not to himself, but to the Medical Association of the State from whence he came. Thanking them for the compliment and the cordial reception, he was invited to a seat in the Association.

A series of resolutions passed by the Association of Superintendents of Insane Asylums, held in Canada within the past year, was read, recommending the establishment of a chair on mental diseases and medical jurisprudence, independent of the chair of physiology, in the several medical colleges of this country—when, on motion, the same was indorsed and adopted by the Association.

The Secretary presented several accounts, which, on motion, the Treasurer was ordered to pay.

The Chairman of the committee on the proposed new Constitution, Dr. Kumpè, sent up his report, upon which some discussion arose—participated in by Drs. Weatherly, Cochran, Lynch, and others—when, on motion of Dr. Ketchum, the further discussion of the subject was deferred until the second day of the next annual session of the Association.

On motion of Dr. Means, Dr. Love was granted the floor to present to the Association copies of the ATLANTA MEDICAL AND SURGICAL JOURNAL, together with its claims to the support of the Southern medical profession.

Dr. Love occupied the attention of the Association for a few moments, presenting specimens of the JOURNAL, and extending its pages to the body for any communications they might desire to insert or send out to the profession through that medium.

On motion, the Secretary was directed to furnish Dr. Love the minutes of the proceedings of the Association, for publication in the ATLANTA MEDICAL AND SURGICAL JOURNAL.

Dr. Berger called the attention of the Association to the system, prevalent in many portions of the country, of making contracts for doing the practice of plantations by the year, and asked the views of the body as to the ethical propriety of the same. This gave rise to some discussion.

Dr. Osborn stated that he had ever been opposed to contracts under former existing circumstances, but was of the opinion that, at the present time, there were many circumstances which would tend to excuse, to some extent, the practice, and he was disposed to regard it with more leniency than formerly.

Dr. Sadler contended that the profession should stand firmly to their Code. He illustrated, by several cases in point, of a commonplace, practical character, that it was not only the duty of the profession, but equally their interest, to stand to their code; that the landlord or planter had as much right to claim that the physician should feed his stock, or keep in repair his wagons, plows, or agricultural implements, and take the chances of getting his pay after the landlord's liens were settled, as to demand that the medical attendant should keep his employees in a condition of physical or physiological repair, and take the chances for remuneration. The landlords are protected by liens, which have been refused to the profession, and the only hope is for the profession to unite for the protection of themselves. If they will do this, and act in concert, the planters will find it to their interest to keep their labor in repair, as well as their implements; and though they may kick against it at first, they will in the end consult their own interest, and become responsible to the physician for his fees for services rendered. Such a course of policy had been pursued by the North Alabama Medical Association; and, though there was some trouble at first, the firmness of the profession had forced the landlords to respect their rights, and foot the bills for services rendered their emyloyees.

Dr. Weatherly presented the Code of the American Medical Association, and contended for its enforcement, in some well-timed and well-directed remarks.

Dr. W. C. Jackson participated in the discussion, and introduced a resolution reaffirming the Code of the American Medical Association.

Dr. Jerome Cochran offered a substitute for Dr. Jackson's resolution, which was in substance as follows:

*Resolved*, That this Association acknowledge and indorse the Code of Ethics of the American Medical Association as the fundamental law of this Association; and deeming any further legislation on this question inexpedient at this time, would refer the matter to the subordinate medical societies of the State, to take such action in the matter as in their judgment may be deemed most expedient.

Dr. Jackson's motion was withdrawn, and the substitute of Dr. Cochran adopted.

A communication was received, extending an invitation to the Association and visiting members of the medical profession to attend a reception to be given by the physicians of Madison county, at the Huntsville Hotel, Wednesday evening, at half-past seven o'clock.

A communication, accompanied with blanks, was received and read—from Dr. Culbertson, of Dayton, Ohio—requesting reports of results of operations of resection, for the purpose of making up reliable statistical reports as to results.

Reports on the diseases and surgery of the different counties being in order:

*From Colbert County.*—Dr. Abernathy presented, through Dr. Despretz, a report on diseases, which was read, and referred to the Committee on Printing.

*From Greene County.*—Dr. H. B. Robinson presented, through the Secretary, a report on diseases, which was read and referred.

*From Jackson County.*—Dr. Bankson was granted further time to make out his report.

*From Jefferson County.*—Dr. Prince presented, through Dr. M. H. Jordan, a report, which was read and referred.

*From Lauderdale County.*—Dr. Jas. Kyle presented a report, through the Secretary, which was read and referred.

*From Laurens County.*—Dr. Sadler applied for permission to report four special cases for the "Transactions," to be referred to the Committee on Printing, which was granted.

*From Madison County.*—Dr. G. D. Norris presented a very interesting report on the topography and diseases of Madison county, which was read by himself, and referred under rule.

In this report, Dr. Norris presented, with many other points, one which was new, and not without especial interest in a practical point. He stated that during the prevalence of small-pox in Huntsville, certain families, at the instance of some one unknown, had resorted to the free use of the *tea of the cimicifuga racemosa*, or black snake-root of the United States Pharmacopœia (*cohosh*), *as a preventive of small-pox.* In the families using the cimicifuga, there occurred no case of the small-pox, though some were exposed to the disease. In the same families, Dr. Norris vaccinated the members, but without effect so long as they continued the use of the cohosh; after ceasing to use the tea as a prophylactic, he again vaccinated them, when the specific effects of the vaccine virus were produced. He submitted the results in these cases as new, and not without interest to the profession. Should further investigation prove that the medicine does possess the power here ascribed to it, the remedy may prove of inestimable value in many cases, to which it is not necessary now to refer.

The Association then adjourned until ten o'clock to-morrow morning.

---

HUNTSVILLE, ALABAMA, March 28, 1872.

The Association met pursuant to adjournment, and was called to order by the President, Dr. Osborn. Minutes of yesterday's proceedings read and approved.

A communication from the President of the Memphis and Charleston, and other railroads, was read, extending the courtesies of their roads to the members traveling by their several routes.

The committee appointed on the purchase of the Marine Hospital reported that the United States Government did not desire to dispose of the Hospital, which report was received, and the committee discharged.

Dr. Heustiss, from the committee on the death of Dr. Miller, submitted his report, which was received and adopted, and the committee discharged.

Dr. Jerome Cochran presented the report of the Chairman of the Committee on the State Board of Health, favoring the plan of Dr. Cochran.

Dr. Means submitted a report from the majority of the committee, favoring the report or position taken by Dr. Weatherly.

Some discussion ensued, participated in by Drs. Weatherly and Means, and replied to by Dr. Cochran.

Dr. Anderson called for the question, when it was decided in favor of the plan submitted by Dr. Cochran, and the committee was discharged.

Dr. Bassett, Chairman of the Committee on the President's address, submitted the following report, which was received and adopted:

Your Committee, to whom was referred the President's address, after careful examination, beg leave to report:

That portion relating to petitioning the Legislature to pass a bill granting to physicians a lien for their fees, next to landlords' and laborers' liens, for services rendered, and the repeal of that section of the *Revised Code* exempting certain property from execution, meets with their approval, and they advise that the Association take such steps as will be most likely to secure its passage by the Legislature.

We heartily concur with the President's suggestions concerning private students, and advise that the Association recommend that the profession throughont the State adopt and enforce the practice of examining students; and if they be found deficient in education, refuse to receive them.

We approve of the formation of District Associations, and advise that the State Association recommend it to the local societies.

We suggest that the President be requested to present his views on the history and periodicity of the great malarial epidemics, at the next annual meeting.

We commend to the attention of this body the President's suggestions in regard to the dispatch of business.

Dr. Despretz presented a plan for the "Alabama Medical Benevolent Aid Society," together with printed copies of by-laws for the government of the same.

On motion of Dr. Ketchum, the plan was indorsed by the Association.

A call for reports from the several counties being in order:

*From Madison County.*—Dr. Dement presented, through Dr. Bassett, an interesting report on surgery, which was read and referred.

In this very able report, was presented the history of a case of resection of the lower end of the humerus, in a young lady, resulting in recovery, wherein the patient retained the power of *flexion, with supination and pronation of the forearm.* Many other cases of interest were presented in this report, as will appear in the published "Transactions."

*From Jackson County.*—Dr. Bankston presented a concise report, which was read and referred under rule.

Prof. T. O. Summers, M.D., of the Southern University, at Greensboro, Alabama, presented, by invitation, a quantitative analysis of the urine in a case of hemorrhagic malarial fever,

Dr. Summers, in his remarks on the paper presented, referred to the physiological explanation of the changes in the phosphates and sulphates, and the production of albumen, given by Dr. Love in a clinical lecture before the class of the Atlanta Medical College, and published in the MEDICAL AND SURGICAL JOURNAL for July, 1871.*

Dr. Sadler expressed himself as feeling a deep interest in anything which would tend to throw light on the pathology and treatment of this disease; that he had found it formidable and fatal in tendency, and that the four cases he had asked permission to report were of this nature. He advocated the use of ergot, to control the hemorrhage, as the proper plan of treatment.

Dr. Taylor, of the United States Army, and Dr. Love, of the Georgia Medical Association, were called upon to express their views upon the subject of the pathology and treatment of the disease.

*In reply to a letter from Dr. Love, requesting a report of his remarks, Dr. Summers furnishes the following:—

"You will notice, gentlemen, in considering this analysis, a remarkable decrease in the earthy phosphates—there being only 3.45 parts to the 1.000 in this urine, whereas normal urine contains about 14. Another suggestive point is, that the quantity of albumen, as shown in the analysis, nearly balances this deficiency in the phosphates.

"In a clinical lecture on 'Albuminuria,' delivered by Prof. Abram Love, of the Atlanta Medical College, I notice that he has regarded the diminution of the salts in this disease as indicative of the 'non-combustion of the albuminoids.' Certain it is that the nervous tissue has not been properly oxydized, or these salts would appear in the urine.

"Dr. Love, in the lecture referred to, has accounted for the non-oxydation of the nitrogenous materials in the liquor sanguinis by the improper arterialization of the blood; and he goes on to say that, as a consequence of this, 'the production of urea is diminished on the one hand, while albumen is eliminated on the other.' I am not prepared as yet to pronounce definitely upon this, as I have not satisfied myself as to the etiology of the disease by the investigations which I have made upon it; but reasoning *a posteriori* from the analysis which I have given you, it does seem to me a natural conclusion. Under no other hypothesis, can I account for the presence of a quantity of albumen so nearly equal to the deficient salts.

"The patient upon whom these investigations were made was treated in accordance with the indications set forth in this analysis, and the result was a perfect recovery."

Dr. Taylor, in his remarks, adverted to the sudden changes in the blood, and the sudden appearance of the hemorrhage. Stated that he had found, in the blood recently drawn from the arm, molecular matter, and vibriones showing rapid motion—some *en rule*, others independent, as seen under the microscope. He supposes that the changes in the blood take place before the blood enters the kidneys. Dr. Taylor states that though he had used the usual tests for bile—such as Petenkofer's, the nitric acid, etc.—he had never been able to detect any trace of bile in the urine; that he had found the blood-corpuscles sometimes crenated, at others broken down, and the coloring matter diffused through the *liquor sanguinis*, in conjunction with molecular elements. He regarded the disease as one of malarial origin, occurring in the general spread of malarial fever.*

Dr. Cochran made the point, that without fermentation or decomposition, vibrious or molecular matter did not make its appearance in fluids, and that it evidenced the absence of oxygen—that vibriones could not exist in conjunction with oxygen. Made the point as a chemical fact well established.

*In reply to a letter, requesting a report of his remarks, Dr. Taylor writes as follows:

"Thomas Barracks, Huntsville, Alabama, April 10, 1872.

"*Dr. Wm. Abram Love, Atlanta, Georgia:*

"Dear Doctor,—Your favor of the 8th instant, in relation to my desultory remarks before the Alabama State Medical Association on the subject of malarial hæmaturia, is received, and I hasten to reply as requested.

"My microscopic examinations were limited to the blood and urine of the patients after the disease had been fully developed, and after death. The magnifying power employed ranged from two hundred and fifty to five hundred diameters. These investigations embraced several specimens of urine,—obtained, in one case, once daily—in another, twice daily—in the others, only a single specimen from each. The time elapsing after the fluids were obtained from the patients, before examination, ranged from about ten minutes to twelve hours.

"In all the specimens of urine examined, I found blood-corpuscles. In one case, when the urine was nearly of the natural color, and when, from the general improved condition of the patient, the opinion was entertained that the hemorrhage had ceased, I found the red blood-corpuscles still present, though in diminished numbers, indicating that the improvement was more apparent than real.

"In the examination of the urine, the following microscopic elements were observed:

"1. The usual epithelial cells of the urinary passage.

Dr. Love, of Georgia, while agreeing in the main with Dr. Taylor as to the results of microscopic investigations of the disease of the blood and urine, as presented in such cases, regarded the disease as one of malarial origin in a broken-down system—broken down under repeated attacks of intermittent or some form of malarial fever. He looked further for the true pathology of the disease—to the minute microscopic anatomy of the lion, as presented by Dr. H. D. Schmidt, of New Orleans. He (Dr. Love) regards the disease as one originating in a *regurgitation,*

"2. A very few hyaline uriniferous tubules.

"3. Vibriones—consisting, apparently, of a row of cells 1-10.000 in diameter—varying from two in number to fifteen or twenty, and enclosed in a delicate tubular investing membrane. They moved with considerable rapidity across the field of vision, and seemed to multiply by subdivision. In one case, a vibrio, consisting of fifteen or twenty cells, separated in the middle, and the respective parts moved with equal facility in opposite directions.

"4. Active molecular elements of cellular structure—ranging from about 1-10.000 to 1-15.000 of an inch in diameter—having slight locomotive powers, so that they could change their focal distances tolerably quickly, but moved their position across the field slowly, and this chiefly by a process of rotation or amoiboid movement; others spun around upon their own axes with great velocity.

"5. Bacteria. Some of these were attached to the red blood-corpuscles. They appeared as caudate moniliform appendages, with slight wavy motions—their length exceeding four or five times the diameter of the blood discs.

"White blood-corpuscles were present in about the usual proportion of malarial blood—that is, rather in excess of what is seen in the blood of vigorous healthy subjects.

"In one case—being that of a child about three years old—the blood was disintegrated, and the coloring matter diffused throughout the fluid, the corpuscles appearing shrunken and crenated. (Hæmaturia.) The examination of the blood was made two hours after death, and embraced a single specimen. It was fluid when drawn, but coagulated after the lapse of a short time. The specimen was obtained from the median vein of the forearm, by making an opening into the same, drawing the blood into a capillary tube, and then placing this under the microscope in about fifteen minutes. In this, the same microscopic blood-changes were observed as in the urine a few hours before, with the exception that there was a much larger proportion of leucocytes in this blood from the arm than in that coming through the kidneys; in fact, these bodies appeared in the proportion of about one to eight or ten of the red corpuscles. This excess may be due in part, perhaps, to the greater facility with which the red blood discs move with the currents setting in from the remote capillaries to the larger interior veins, after death; but, I apprehend, not wholly so. The vibriones were made up of two or three cells only, but so to speak, *of the bile from the minute biliary tubules into the anastomosing, minute lymphatic vessels of the liver*—passing thence along the lymphatics, to be poured into the thoracic duct, thence into the venous system and the general circulation. This he believes to be the origin of the *blood-poison* in the disease, and the peculiar character of the blood referred to by Dr. Taylor and others in the microscopy and chemistry of the blood and urine in such cases. He regards the disease as one of blood-poison from the direct introduction of the biliary secretion into were very active; the single molecular cell-structure rotated very rapidly, and the bacteria, though less in size, were still present—a few attached to the red blood-corpuscles, while others were not.

"The urine of all the patients was examined at various times for the presence of bile; but in none did it appear in response to the nitric acid, Heller's or Petenkofer's tests. These conditions of the blood indicate its incipient decomposition, and perhaps imperfect aeration, and that these elements are developed within the body before death by the zymotic changes incident to the malarial poisoning. In one instance, the urine had remained in the bladder less than three-quarters of an hour, and yet these zymotic changes and active elements were abundantly manifest. This was observed the day before the patient died, showing that the blood from the kidneys was a fair representation of the condition of that fluid throughout the body at the time.

"My remarks in relation to the prevalence of the malarial fevers in high northern latitudes were in effect to show that the prevalent opinion entertained by the profession as to their limitation is erroneous. It has been said, repeatedly, that these fevers are not found in the Lake Superior country, in Northern Minnesota, and in the territories westward therefrom. My own observations are the very contrary of this; for I have seen native white and Indian inhabitants on the north shore of the lake, half-breeds from Hudson's Bay, and white inhabitants from Lake Winnepeg, who had never been south of the southern shore of Superior, who had well-marked forms of ague, even in the colder months of the year, and I have been credibly informed that the valleys of the Sascathawan and Assinaboin, autumnal fevers, which are of the remittent type generally, are very severe, often fatal, and require the administration of quinine in large doses for their cure. It is true that the remissions are not always as thoroughly pronounced as in the sections farther south, but they are not to be mistaken by any careful observer. Indeed, the native Indians recognize them, and have their special modes of practice for their relief from disorders of this kind.

"Whether these remarks are pertinent to the subject you wish to elucidate, I do not know; but as they bear on the subject of malaria, and do not seem to be generally known, I send them for your use, if you wish.

"Yours, truly, M. H. Taylor,

"Assistant Surgeon United States Army."

the blood through the lymphatics of the liver. The color or *bronze* condttion of the skin and tissues may result from the additional action of ammonia, or a compound of nitrogen and hydrogen in the blood, acting on the non-assimilable albumen in the *liquor sanguinis.*

His treatment is based upon the pathology of the disease. Much may be expected from the antizymotic action of sulphurous acid gas introduced or liberated in the stomach by the introduction of sulphate, or preferably hyposulphite, of soda, thrown in small and oft-repeated doses into the stomach. (Chloroform, in small doses by the stomach, alternated with the hyposulphites, will be found of much service.) Calomel he has not found very advantageous. Quinine, followed by the hyposulphites, to anticipate the paroxysms, opium at night, and when the paroxysms have been arrested, the nitric and muriatic acids as tonics, give the leading indications in treatment. Saline baths or sponging, or the sponging with nitro-muriatic acid in a diluted form, or the chlorinated soda, will often promote cutaneous action as well as renal, and allay the nettle-rash so frequently annoying in the convalescence.

The paper of Dr. Summers was received, and referred to the Committee on Printing.

*From Marengo County.*—Dr. George Whitfield presented a report, which was received and referred.

*From Mobile County.*—Dr. G. A. Moses presented a report on the topography and diseases of Mobile, with a map, which was received, and referred under rule.

Dr. J. T. Gilmore presented an elaborate report on the surgery of Mobile, and gave, verbally, a synopsis of the same, which was well received. He presented many interesting cases, to which we would gladly allude, but for want of space and our inability to do the report justice in presenting a brief synopsis.

Dr. Gilmore believes, with Dr. Nathan Smith, that chloroform and chloral prove injurious to the system in surgery, and tend to the production of gangrene and pyæmia, as well as to secondary hemorrhage. In cases of secondary hemorrhage, Dr. Gilmore believes that the occurrence of the hemorrhage is always preceded by a hemorrhage *guttatim;* and in such cases, he resorts to the *perchloride of iron*, inserted into the wound at the point of the *guttatim* hemorrhage *by means of a hypodermic syringe*. This, he asserts, will often prevent the necessity of opening the wound and ligaturing the bleeding vessel.

Dr. Gilmore, in the treatment of suppurating wounds, does not hold carbolic acid in such high esteem as many of the profession; he does not regard it as possessing claims very far superior to other disinfectants. He places a high estimate on the use of oakum for surrounding suppurating wounds, and earnestly recommends its use to the profession.

In his report, he gives an interesting case of bone denuded of its periosteum, where, after the bone was removed, it was reproduced by the periosteum. He also reports a case of reduction of long-standing dislocation of femur, by manipulation, precedented only by the case of Dr. Sayre, and terminating favorably under the plan pursued at Bellevue Hospital.

It would afford us pleasure to follow Dr. Gilmore through with his synopsis, but want of space forbids.

Dr. W. H. Anderson read a very interesting report on the sanitary condition of Mobile, in which he speaks in a very complimentary manner of the effects of Dr. Cochran's system of drainage, and his hygienic measures generally. Referred to Committee on Printing.

Dr. Gaines requested further time to prepare his paper on phthisis pulmonalis, which was granted.

Dr. Mastin submitted a very elaborate essay on genito-urinary surgery, which he read in part, presenting a very clear and concise account of the present acknowledged views of the profession on the subject, together with many original points, which, it is hoped, will be given to the profession in the future.

*From Montgomery County.*—Dr. J. H. Blue sent up a written report, which was received, and referred to the Committee on Printing.

*From Elmore County.*—Dr. E. Mason submitted an obstetric record, which was received, and referred under rule.

*Contributions.*—Dr. A. G. Mabry, of Selma, submitted "A Reply to Dr. T. C. Osborn on the Use of Quinine in Malaria," which was received, and referred to the Committee on Printing.

Dr. Crawford, of Bibb county, asked further time in which to complete his report on the diseases of Bibb county, and its reference to the Committee on Printing, which was granted.

Dr. Lynch, of Wilcox county, desired that the records of the "Transactions" be corrected, in the particular that he was not correctly reported by the Secretary in saying that he and another professional brother had administered *chloral* in one case without sedative effects.

Dr. T. C. Osborn presented to the Association a tooth from a child thirteen days old; also one from a lady aged sixty-one years—the latter being of recent growth.

On motion of Dr. L. H. Sadler, the following resolution was adopted:

WHEREAS, It is a pleasant custom of State Medical Associations, in some States, to send greetings and delegates to neighboring State Associations;

*Resolved*, That the Alabama State Medical Association appoint one delegate to attend the meetings of the State Medical Association of Tennessee and Georgia, which will take place in a few days in those States, and the delegates be authorized to extend the cordial wishes of this Association for their continued prosperity.

On motion, Dr. T. C. Osborn was appointed a delegate to the State Medical Association of Tennessee, and Dr. T. A. Means a delegate to the State Medical Association of Georgia.

The Association then proceeded to the election of officers for the ensuing year, which resulted as follows:

*President*—Dr. G. E. Kumpè, of Leighton, Alabama.
*First Vice-President*—Dr. E. P. Gaines, of Mobile, Alabama.
*Second Vice-President*—Dr. C. A. Crow, of Mobile, Alabama.
*Third Vice-President*—Dr. L. S. Bonner, of Reform, Alabama.
*First Recording Secretary*—Dr. Jerome Cochran, of Mobile, Alabama.
*Second Recording Secretary*—Dr. T. O. Summers, of Greensboro, Alabama.
*Corresponding Secretary*—Dr. John Little, Jr., Tuscumbia, Alabama.
*Treasurer*—Dr. W. C. Jackson, of Montgomery, Alabama.
*Orator*—Dr. M. H. Jordan, of Elyton, Alabama.
*Valedictorian*—Dr. L. H. Sadler, of Leighton, Alabama.

Place of next annual meeting—Tuscumbia, on the last Tuesday in March, 1873.

The Association adjourned, after passing a vote of thanks to the outgoing officers, the railroads, the medical profession of Madison county, and citizens of Huntsville for kindly attentions.

